# Incisional hernia as an unusual cause of hepatic encephalopathy in a 62-year-old man with cirrhosis: a case report

**DOI:** 10.4076/1752-1947-3-7315

**Published:** 2009-09-17

**Authors:** Muge Ustaoglu, Tulay Bakir, Ahmet Bektas, Osman Cure, Bulent Gungor

**Affiliations:** 1 Department of Gastroenterology, Ondokuz Mayis University, Faculty of Medicine 55139 Samsun Turkey; 2 Department of Internal Medicine, Ondokuz Mayis University, Faculty of Medicine 55139 Samsun Turkey; 3 Department of General Surgery, Ondokuz Mayis University, Faculty of Medicine 55139 Samsun Turkey

## Abstract

**Introduction:**

Hepatic encephalopathy may be initiated by many factors such as gastrointestinal bleeding, infections, fluid and electrolyte disturbances. Hypokalemia is one of the most commonly encountered electrolyte abnormalities causing hepatic encephalopathy in patients with cirrhosis.

**Case presentation:**

We present the case of a 62-year-old Caucasian man with decompensated liver cirrhosis having multiple episodes of hepatic encephalopathy precipitated by vomiting. He had an incisional hernia at the right lumbar region. A barium contrast study of the small intestine and magnetic resonance imaging showed that the hernial sac included gastric antrum and bowel. We observed that hepatic encephalopathy coincided with hypokalemia as a result of a large volume of vomiting triggered by the collapsed hernial sac. Hepatic encephalopathy was resolved by administration of intravenous potassium.

**Conclusion:**

This case illustrates that a hernia causing a large volume of vomiting may be a precipitant factor in the development of hepatic encephalopathy.

## Introduction

Hepatic encephalopathy (HE) or portal systemic encephalopathy is a complex neuropsychiatric syndrome associated with either acute or chronic liver failure. The symptoms of HE range from altered sleep patterns to stupor and deep coma [1]. HE is precipitated by a number of factors such as gastrointestinal bleeding, infections, fluid and electrolyte disturbances, constipation, excessive dietary protein, use of sedatives and creation of a surgical shunt or the placement of a transjugular intrahepatic porto-systemic shunt [2]. Hypokalemia is one of the most commonly encountered electrolyte abnormalities causing HE in patients with cirrhosis. We present the case of a patient with episodes of HE and hypokalemia induced by vomiting.

## Case presentation

A 62-year-old Caucasian man was diagnosed with decompensated liver cirrhosis secondary to hepatitis C virus infection in 2002. He was hospitalized because of HE several times during 2004.

**Figure 1.**
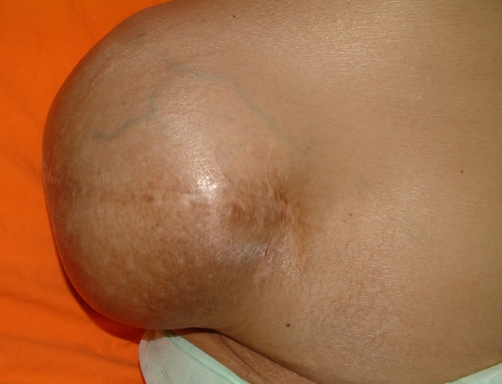
Photograph showing the patient with an incisional hernia.

In November 2004, the patient was admitted to the emergency department because of personality changes that developed four hours after a lot of vomiting. After admission, loss of consciousness and respiratory distress occurred. He had a history of surgical operation to the right kidney due to nephrolithiasis approximately 29 years previously. Medications before admission included propranolol, aldactone, lactulose and ursodeoxycholic acid. Physical examination on admission indicated a blood pressure of 100/70 mmHg, heart rate 68 beats/minute and respiratory rate 24/min. There was mild jaundice, fetor hepaticus, splenomegaly (3 cm below costal margin) and a hernial sac about 20 cm in diameter at the right lumbar incision, reducible with difficulty (Figure 1). After administering first aid, the patient was transferred to the internal medicine ward.

Admission laboratory tests were as follows: hemoglobin 11.6 g/dl, leukocytes 4,000/mm^3^, platelets 49,000/mm^3^, sodium 133 mEq/l, potassium 2.5 mEq/l, glucose 115 mg/dl, creatinine 0.7 mg/dl, alkaline phosphatase 220 U/L, aspartate aminotransferase (AST) 18 U/L, alanine aminotransferase (ALT) 30 U/l, γ-glutamyl transpeptidase 20 U/l, total bilirubin 4 mg/dl, direct bilirubin 2.1 mg/dl, total protein 5.7 g/dl, albumin 2.2 mg/dl, activated partial thromboplastin time 25 sec, prothrombin time: 15 sec, and international normalized ratio (INR) 1.2. The plasma ammonia level on admission was 422 μg/dl (normal range 25 to 94 μg/dl). His Child-Pugh score was 10 (Child’s class C). The model for end-stage liver disease (MELD) score was 14.

Nasogastric suction was performed and approximately 2,000 ml dark green bile aspirated. The patient received intravenous 40 mEq of potassium chloride (at a rate of 20 mEq/hour) over a period of 2 hours in the emergency department. Thereafter, intravenous potassium supplements in saline and in dextrose solution were given and an enema containing lactulose and ampicillin was given twice a day for 10 days. The patient recovered consciousness after the correction of the hypokalemia. Subsequently, the previous drug therapy was re-established.

**Figure 2.**
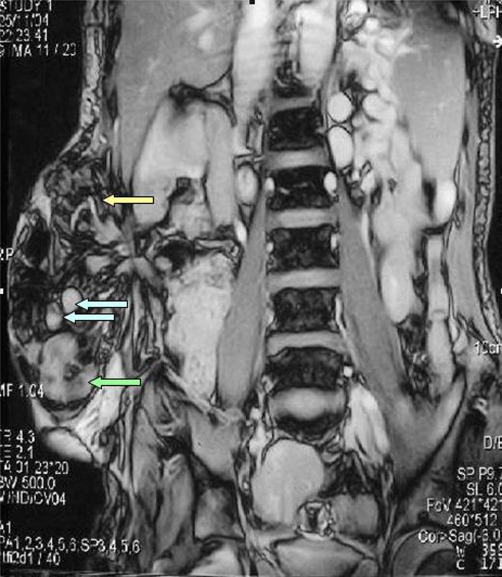
Magnetic resonance image of the abdomen showing a hernial sac containing gastric antrum (green arrow), segments of small intestine (blue arrow) and mesenteric fatty tissue (yellow arrow).

**Figure 3.**
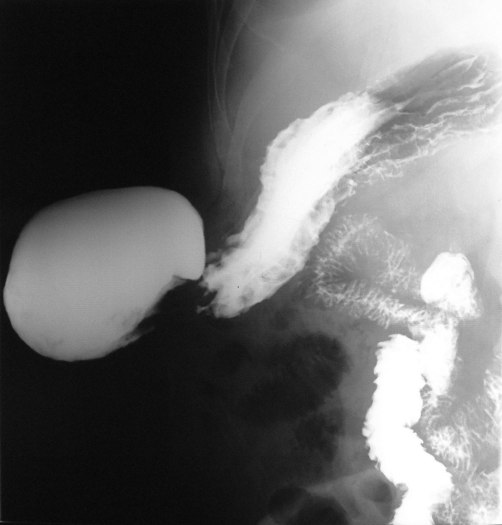
Barium study showing a hernial sac containing gastric antrum, duodenum and proximal jejunum.

Plain abdominal radiography taken in the upright position demonstrated no air-fluid levels, suggesting small bowel obstruction. Abdominal ultrasonography showed parenchymal inhomogeneity of the liver with irregular margins, splenomegaly (with a craniocaudal diameter of 157 mm), a large splenic vein, and a hernial sac sized 21 × 13 × 9.5 cm located in the right lumbar region. Magnetic resonance imaging revealed a 6.5 cm fascial defect and mesenteric fatty tissue and bowel as the content of the hernial sac but with no sign of incarceration (Figure 2). A barium-contrast study of the small intestine showed that the hernial sac contained gastric antrum, duodenum and proximal jejunum (Figure 3). Upper gastrointestinal endoscopy revealed straight, small-sized (F1) varices over the lower third of the esophagus and food retention, despite the patient fasting for at least 12 hours. Furthermore, a decentralization and deviation of the pylorus and antrum were observed.

The condition of the patient was discussed with the general surgeons. Repair of the hernia was not recommended because of the high risk of general anesthesia and operation and the high rate of postoperative mortality in patients with cirrhosis.

After two weeks, we observed a second episode of HE precipitated by a large volume of vomiting (approximately 2000 ml). Hypokalemia was again evident, and the patient lost consciousness, necessitating intravenous potassium administration. Three similar episodes were observed during the hospitalization period. During all of these episodes, his serum potassium concentration fell rapidly following a large volume of vomiting and the hernia sac collapsed. When the hernia sac was reduced, copious amount of bilious fluid flowed from the nasogastric tube.

Table 1 shows the neurological and laboratory findings of all hepatic encephalopathy episodes observed during the hospital stay.

**Table 1.** Neurological and laboratory findings of all hepatic encephalopathy episodes observed during the patient’s hospital stay

Parameters		Hepatic encephalopathy episodes				
		1^st^	2^nd^	3^rd^	4^th^	5^th^

Neurological findings after vomiting		Somnolence	Confusion	Disorientation	Slurred speech	Lethargy, asterixis
Serum sodium (mEq/l)	Before vomiting	NA	138	143	139	135
	After vomiting	133	129	136	130	129
Serum potassium (mEq/l)	Before vomiting	NA	3.8	3.9	4.0	4.2
	After vomiting	2.5	2.7	2.8	3.0	3.1
Plasma NH_3_ (mcg/dl) after vomiting		422	374	360	362	381

NA, not available.

The patient was discharged 10 weeks after admission and he died 15 months later.

## Discussion

In patients with cirrhosis, hypokalemia may be affected by many factors such as vomiting, diarrhea, malabsorption, use of diuretics and/or cathartics, secondary hyperaldosteronism and poor oral intake [3]. Hypokalemia is a consequence of voluminous vomiting causing the loss of potassium in the vomitus, and also secondary hyperaldosteronism due to hypovolemia [4]. Hypokalemia and concurrent alkalosis increase the production of ammonia in the kidneys [5], and both of these factors may also contribute to the conversion of ammonium (NH_4_^+^) into ammonia (NH_3_) which can cross the blood-brain barrier [6]. Ammonia has been considered the most important causative factor in the pathogenesis of HE. The principle of treatment of hypokalemia-induced HE is the correction of the potassium deficiency and the treatment of the factors that cause hypokalemia.

The survival of patients with liver cirrhosis is highly variable since it is influenced by many factors such as gastrointestinal hemorrhage, infections, HE and hepatocellular carcinoma. In a recent study, the feasibility of survival of decompensated cirrhosis patients was 81.8% and 50.8% at 1 and 5 years, respectively [7]. Some scoring systems such as Child-Pugh and MELD score have been used to predict survival of patients with cirrhosis based on clinical information and laboratory results. The Child-Pugh corresponds with survival. The reported 1-year survival rates of Child’s A, B and C cirrhosis patients are almost 100%, 80% and 45%, respectively [8]. The 5-year survival rates of Child-Pugh Class A, B and C were 69.6%, 46.3% and 36.4%, respectively [7]. The MELD scoring system is a reliable disease-severity index and is an accurate predictor of short-term survival for patients with liver cirrhosis. Three-month survival rates for a patient waiting for a liver transplant with MELD scores of up to 15 points, scores of 30 points and of 40 points are approximately 95%, 65%, and 10% to 15%, respectively [9]. However, in clinical practice, the MELD score should not be used to predict long-term survival [10]. Although developing HE affects patient survival independent of the MELD score, an association between MELD score and HE, as well as HE and mortality, are asserted. HE is an important complication of decompensated liver cirrhosis, and it is associated with shortened survival. In addition, the poorer prognosis of patients with cirrhosis and HE has been reported in male patients, patients with increased serum bilirubin and alkaline phosphatase levels.

Abdominal wall hernias are commonly seen in patients with cirrhosis and ascites [11,12]. The main causative factors for hernia development in patients with cirrhosis are increased intra-abdominal pressure and muscular wasting due to malnutrition [13]. Patients with liver cirrhosis, especially those with Child’s B or C cirrhosis, have increased morbidity and mortality associated with anesthesia and surgery [14]. Therefore, surgical treatment of hernias should be considered only if a complication occurs such as incarceration, strangulation, ulceration, rupture or leakage of ascitic fluid [15].

## Conclusion

As we observed in our patient, an incisional hernia containing a part of the stomach and/or the duodenum can cause a large volume of vomiting which may result in intravascular volume depletion and electrolyte imbalance, especially hypokalemia. This condition can precipitate HE in patients with cirrhosis. The decision for herniorrhaphy in such patients should be made after evaluating the possible benefits and risks of the surgery.

## Abbreviations

ALT: alanine aminotransferase
AST: aspartate aminotransferase
HE: hepatic encephalopathy
INR: international normalized ratio
MELD: model for end-stage liver disease
